# A Comparison of Perceptions of Estonian and Latvian Hunters With Regard to the Control of African Swine Fever

**DOI:** 10.3389/fvets.2021.642126

**Published:** 2021-04-14

**Authors:** Nico Urner, Carola Sauter-Louis, Christoph Staubach, Franz Josef Conraths, Katja Schulz

**Affiliations:** Friedrich-Loeffler-Institut, Federal Research Institute for Animal Health, Institute of Epidemiology, Greifswald, Germany

**Keywords:** African swine fever, participatory epidemiology, control measures, passive surveillance, acceptability, hunter, wild boar

## Abstract

Since the first detected African swine fever (ASF) cases in Lithuanian wild boar in 2014, the virus has occurred in many other member states of the European Union (EU), most recently in Belgium in 2018 and in Germany in 2020. Passive surveillance and various control measures are implemented as part of the strategy to stop disease spread in the wild boar population. Within this framework, hunters perform important activities, such as the removal of carcasses, fencing or hunting. Therefore, the successful implementation of these measures largely depends on their acceptability by hunters. Methods of participatory epidemiology can be used to determine the acceptance of control measures. The use of participatory methods allows the involvement of key stakeholders in the design, the implementation and the analysis of control and surveillance activities. In the present study, two studies that had been conducted using participatory epidemiology with hunters in Estonia and Latvia were compared on the topics recruitment, participants, facilitators, focus group discussion (FGDs) and their contents. The aim was to evaluate similarities and differences in the two studies and to identify a broader spectrum of possibilities to increase the willingness of hunters supporting the fight against ASF. Evaluating all conducted FGDs in both countries showed primarily similarities in the perceptions and opinions of the hunters in Estonia and Latvia. One notable difference was that passive surveillance in Latvia was perceived mostly as topic of duty and ethics rather than an issue driven by incentives. Participatory methods have proven to be an effective tool in the evaluation of the acceptance of established ASF control systems. The results of this study point out further chances for improving the cooperation with hunters in the future. Nevertheless, the importance of gathering and analyzing the opinions of hunters in all ASF affected countries individually is highlighted.

## Introduction

The recent entry of African swine fever (ASF) into Germany in September 2020 showed that the ASF spread in the European Union has not yet come to a hold (1). Since the beginning of the current epidemic in Georgia in 2007, more and more wild boar and domestic pigs have become infected globally (2). The ASF virus emerged in Lithuania, Poland, Latvia and Estonia as the first affected member states in the Eastern part of the EU (3). Currently, there are two main mechanisms, which are deemed to be responsible for the spread of ASF, i.e., trans-regional human mediated virus spread, sometimes over long distances, and local transmission by migrating wild boar (3–5). The potential role of wild boar as a susceptible species in the spread of ASF emphasizes the importance of establishing measures aimed at controlling local wild boar populations (2, 6–10).

Hunters belong to the most important stakeholders in the implementation of ASF control measures in the wild boar population (11, 12). Their regular presence in the forest, their experience and knowledge regarding local wildlife make them valuable partners with regard to control measures and passive surveillance. So far, hunters have been primarily involved in the implementation of mandatory processes, such as wild boar carcass searches, removal of carcasses from the environment and shooting wild boar. However, expert knowledge on the local situation, also with respect to the peculiarities of the wild boar population, is an important basis for the control of the ASF (11, 13). As mentioned by experts, hunters should therefore be included in the decision-making process (2, 14). This can be achieved by using methods of participatory epidemiology (PE) (15, 16). PE allows the involvement of stakeholders, e.g., in data collection or decision making on topics relevant for the community (11, 14, 17, 18). Participatory methods such as focus group discussions (FGDs) in combination with visualization or ranking and scoring tools are widely used in developing countries to support quantitative data generation in rural areas (13, 17, 19–22). Despite its potential in considering issues from different points of view and implementing specific local measures avoiding unpopular approaches, PE has not frequently been used in developed countries so far (17, 23).

To employ the advantages of PE by investigating perceptions of hunters and thus learning more about their motivations or reasons for hindrance to support ASF control in wild boar, two PE studies were conducted in Estonia and Latvia. In both studies, the same methods of FGD and visualization methods were used and regional opinions on the acceptance of ASF control measures and passive surveillance were collected and analyzed (24, 25). In the present study, the results of these two studies were compared, thus assessing similarities and differences. By comparing both studies, we aimed at identifying functioning processes and difficulties (26) in current control strategies against ASF, which may be addressed in future collaboration with hunters to increase the acceptance of passive surveillance and defined ASF control measures.

## Materials and Methods

### Recruitment

Hunters from different areas in Estonia and Latvia were invited to participate. We intended to include a broad range of experiences and perceptions regarding ASF. In co-operation with hunting communities from Estonia and Latvia, leading hunters of regional hunting organizations were contacted. They were informed about PE and the aims of the studies and asked to invite hunters to the FGDs. In Latvia, staff of the “Latvian Food and Veterinary Service” contacted leading hunters. In Estonia, staff of the Veterinary and Food Laboratory contacted potential participants. The Veterinary and Food Laboratory is a facility, to which hunters regularly deliver samples.

### Participants

It was planned to form ten FGDs per country with four to six participating hunters per group. The only requirement for participation was the willingness of the hunters to attend the meetings.

### Facilitators and Focus Group Discussions

The participatory methods used by Urner et al. (24, 25) were adapted from Calba et al. (13) and Schulz et al. (11). The FGDs were divided into two tasks with regard to control measures and two tasks concerning passive surveillance. In each country, they were moderated by a national facilitator. The facilitators' responsibility was to introduce each task to the hunters and explain issues to avoid misunderstandings. In addition, the facilitators had the function to stimulate discussions and encourage reticent participants to express their views while moderating dominant participants. The facilitators were asked not to express their own personal view or to emphasize any particular opinion. The discussions were transcribed in Estonian and Latvian and translated into English.

### Content

#### Acceptability of Control Measures

For the first task, the participating hunters were asked to enumerate all stakeholders they perceived as being part of the ASF control system. Subsequently, they were motivated to indicate the quantity of contacts from hunters to stakeholders and vice versa with four different arrows (no contact, little contact, normal contact, intensive contact). In addition, they were asked to rate the intensity of contacts qualitatively. To this end, each hunter assessed the contacts using smileys as good, neutral or bad (individual ratings). The last step of the first task was that the hunters were asked to use proportional piling to illustrate their trust in the stakeholders with respect to implementing control measures. For this purpose, the participants were given 100 glass beans, which they had to distribute among all stakeholders in proportion to their trust in the stakeholders to implement control measures appropriately (based on a consensus within the group).

In the second task, a list of six control measures was presented to the hunters [fencing, ban of hunting, including professionals for intensive hunting (police/army), increased hunting of female wild boar, incentives for hunting and increased carcass search and removal]. The participants were then asked to list additional measures, they could think of. All control measures were evaluated based on the hunters' satisfaction in implementing them (individual rating using smileys) and on the trust that the implementation of the measure might help to control ASF (consensus within the group, using proportional piling).

#### Acceptability of Passive Surveillance and Different Motivation Options

In the third task, the participants were asked to list positive and negative consequences that came to their mind when finding dead wild boar. Thereafter, the participants had to discuss until they had reached consensus and to evaluate the mentioned consequences by distributing 100 glass beans proportionally to the perceived impact the consequence would have on the hunters (proportional piling).

In the fourth task, four options to increase the motivation of hunters to participate in passive surveillance were presented to the hunters (increase of currently paid incentives, passive surveillance achieving the benefit of reduction of infection pressure in the wild boar population, only reporting dead wild boar without any further work for the hunter and detailed feedback from the relevant authority to the hunter). The participants were asked to add further options. Using proportional piling the hunters had to illustrate the potential of the options to motivate them to increase their engagement in passive surveillance.

### Analysis

The results of the participatory methods were analyzed semi-quantitatively. To this end, the four different arrows were assigned to the numbers 0, 1, 2, 3 and the smileys to the numbers −1, 0, 1. For each option evaluated by these tools (stakeholders, control measures.), the average for all groups was calculated.

To evaluate proportional piling, a weighted average was calculated for each option (Stakeholder, control measures.). To calculate the trust *T*_*SHi*_ for a mentioned stakeholder (a) *SH*_*i*_, the number of stakeholders mentioned in all groups *SH*, the number of groups which mentioned stakeholder (a) *N*_*SHi*_, the number of stakeholders in the group in which stakeholder (a) was mentioned $C_{j}^{SH}$ and the glass beans allocated to stakeholder (a) in each group *GB*_*ij*_ were taken into account. Details are described in Urner et al. (24, 25).

$$\begin{array}{l} T_{SH_{i}}=\frac{1}{N_{SH_{i}}}\cdot \overset{10}{\underset{j=1}{\sum }}\frac{C_{j}^{SH}}{\overset{10}{\underset{j=1}{\sum }}C_{j}^{SH^{\prime }}}\cdot {GB}_{ij}, \end{array}$$

The trust in a control measure to help control ASF, the impact of possible consequences on the hunters and the potential of an option to motivate hunters to participate in passive surveillance were calculated accordingly.

The results of the discussions were included descriptively.

The data and results from both countries were descriptively compared regarding the topics recruitment, participants, facilitators and FGDs.

## Results

### Recruitment

The recruitment of participants were done similarly in both studies. A list of contact persons (leading hunters of local hunting clubs) had been provided by the national hunting organizations. These contact persons were contacted by phone or mail and informed about the aims of the study. The only difference was the organization that had contacted leading hunters of regional hunting organizations.

### Participants

In total, 96 hunters participated, 46 in Estonia and 50 in Latvia. In each country, one woman participated. The age of the participants was no criteria for participation. To respect their personal rights and to keep the FGDs anonymous, they were not asked for their age. The estimated average age was 50 years.

### Facilitator and Focus Group Discussions

Twenty FGDs were organized from May 2019 to July 2019. Ten FGDs took place in each country, with two to seven hunters per meeting. The group size did not differ in the two studies.

In Estonia, the facilitator was a female staff member of the Estonian University of Life Science, who had not worked with hunters previously and had not been involved in ASF control. She participated in a 3-day training school for participatory methods before the PE study started in Estonia. The study design was practiced under the guidance of the supervising author, who received PE training at the French Agricultural Research Centre for International Development (CIRAD) (11). In Estonia, only the facilitator attended the meetings. The discussions were therefore audio-recorded and afterwards transcribed by the facilitator. In Latvia, the facilitator was a female staff member of the Latvian Food and Veterinary Service, who had not worked with hunters previously and had not been involved in ASF control. The Latvian facilitator did not receive formal participatory training, but practiced the procedures during the discussions with the supervising author and the Estonian facilitator. The Latvian facilitator was assisted by two colleagues from the Latvian Food and Veterinary Service. One of them, a male colleague, was present as an observer and provided scientific background for questions regarding wild boar and the other one, a lady, transcribed the discussions. For analysis, the transcriptions were translated into English by the Language Centre of the Estonian University of Life Sciences in Estonia and the professional translator company “Skrivanek Baltic” in Latvia.

### Contents

#### Acceptability of Control Measures

The listings and ratings of the stakeholders involved in controlling ASF of the Estonian and Latvian participants were similar (Table 1). In both countries, the minor contact to the research centers (Estonian University of Life Science and Institute BIOR) was perceived as unsatisfactory. Participants in both countries rated the police and the army as the least trustworthy organizations with one of the lowest contact rates. Several stakeholders in society, such as the media, farmers and animal protection organizations were mentioned only in Latvia.

**Table 1:** The top five stakeholders rated by the participants to be the most trustworthy to implement control measures in an appropriate manner.

**Estonia**	**Rank**	**Latvia**
Hunters	1	Food and Veterinary Service
Veterinary and Food Laboratory	2	Hunters
Hunting Council of a county	3	Hunting organization
Estonian Hunters' Society	4	State Forest Service
Estonian University of Life Sciences (EMÜ)	5	Institute BIOR

**Figure 1:**
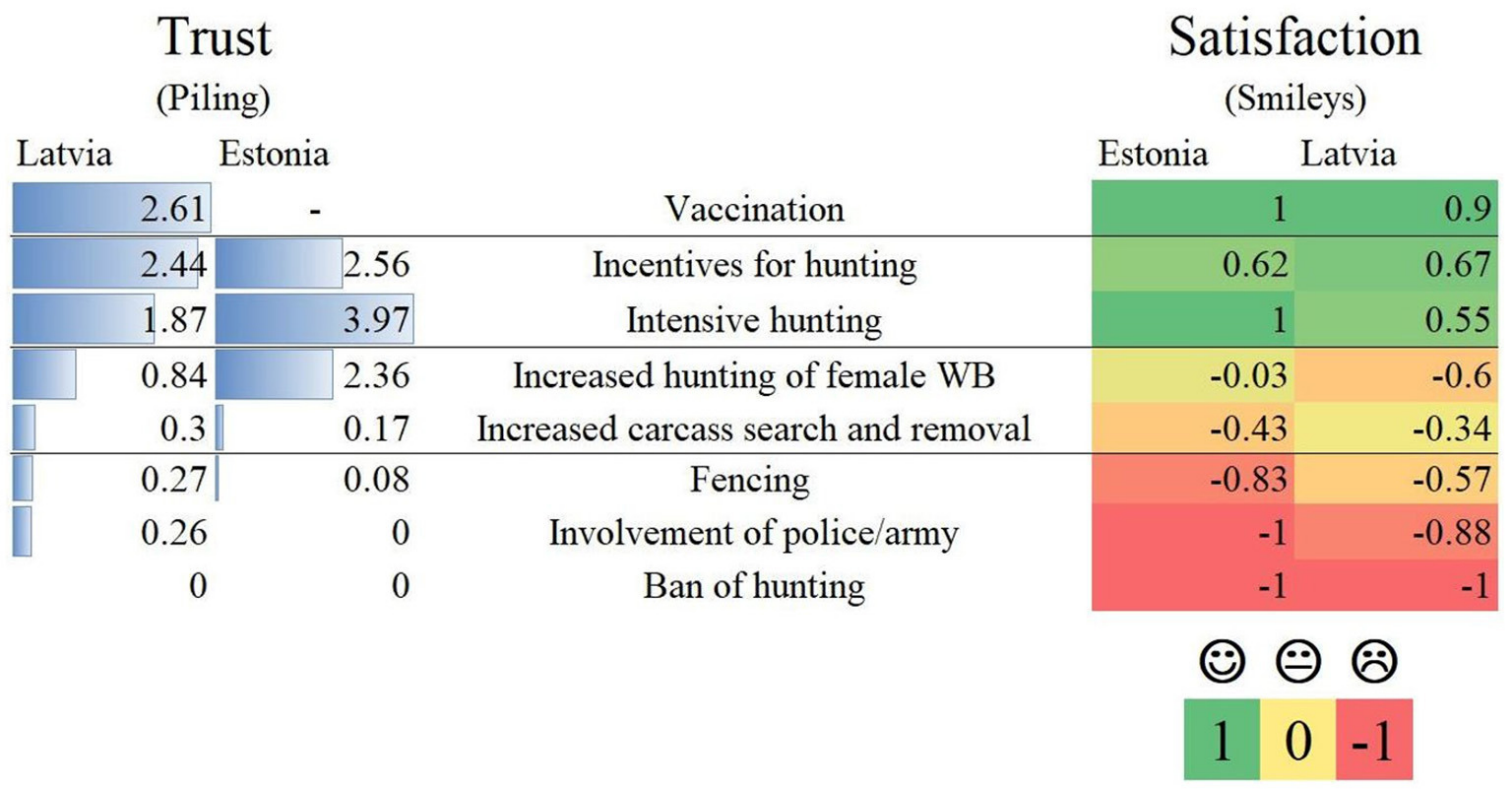
Control measures rated by trust to control ASF and satisfaction in implementing them of Estonian and Latvian participants in ten focus group discussions comparison.

All hunters rated vaccination and hunting as the most trustworthy measures to control ASF and most satisfactory to implement (Figure 1). In Estonia, vaccination was not included in proportional piling by the facilitator as vaccination is currently not an option because there is no functional vaccine (27). Nevertheless, the hunters mentioned in the discussions that they would rate vaccination as the most trustworthy measure. The moral conflict of producing orphans by hunting female wild boar in the farrowing season was mentioned in discussions in both countries. The least trusted control measures in Estonia and Latvia overlapped as well (Figure 1). Similar reasons were mentioned, such as the hindrance of all game animals if a fence is built up. Implementing biosecurity measures during hunting was only mentioned in Latvia. It was trusted mediocre in controlling ASF and perceived satisfactory to implement. On the other hand, various hunting methods were mentioned only by Estonian participants. For example, bait feeding and shooting was highly trusted and considered satisfactory to implement.

#### Acceptability of Passive Surveillance and Different Motivation Options

The perceived consequences of finding dead wild boar overlapped in both countries. However, the assessment of the impact for hunters differed.

**Figure 2:**
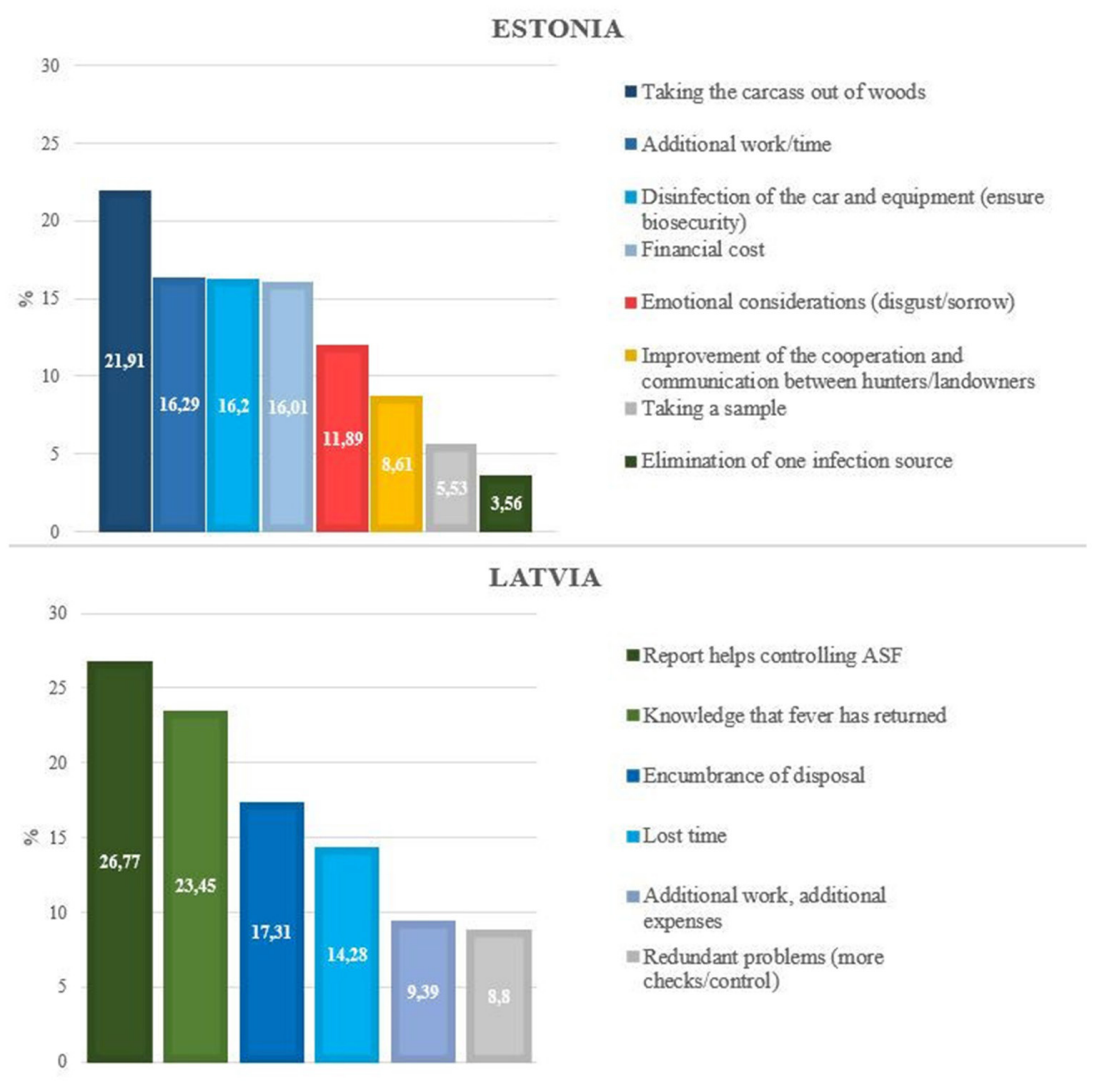
Perceived extent of the impact by hunters of potential consequences when a dead boar is found, expressed as a percentage of all evaluated consequences in Estonia (*n* = 46) and Latvia (*n* = 50). The consequences are colored in green for ethical consequences, blue for consequences on time, work and money and reddish for emotional consequences.

All participants mentioned consequences such as extra work, lost time, financial costs, recovering and disposing of the carcass. In Latvia, the perceived consequences focused on the fact that ASF can be controlled by searching carcasses (and removing them). This was mentioned as the “hunters' duty” in the discussions. In Estonia, the focus was rather on the negative consequences (Figure 2).

**Figure 3:**
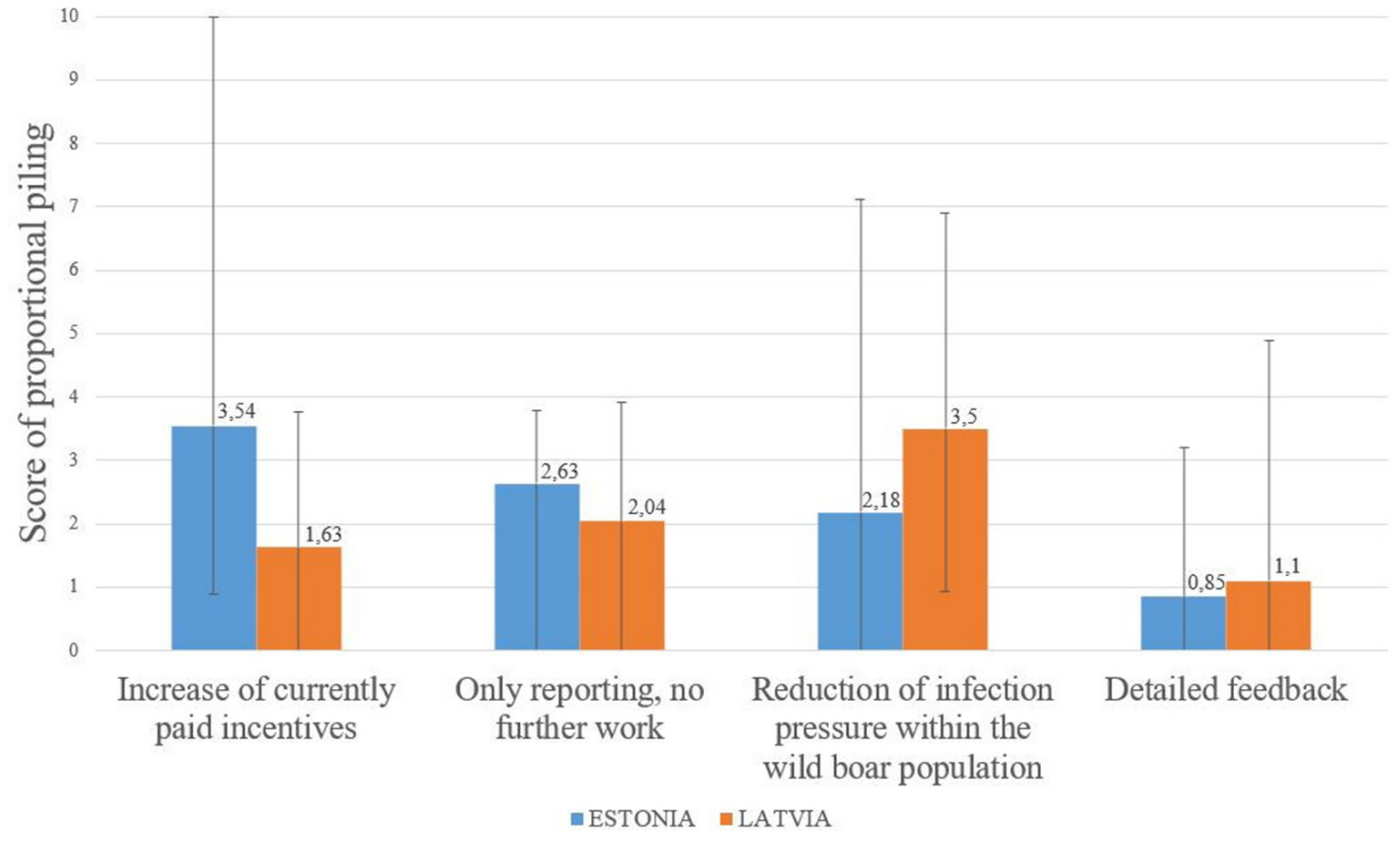
Comparison of the perceived possible effectiveness of tools to increase participation in passive surveillance based on the calculated weighted average of the proportional piling. The average of all groups is displayed along with the range between minimum and maximum value of the weighted piles.

Comparing the proposed options to further increase participation in passive surveillance showed that in Estonia, an increase in financial incentives was considered more motivating than mere reporting with no further work. In Latvia, the pure idea of reducing the infection pressure in the wild boar population by searching for carcasses and removing them was considered the most motivating factor (Figure 3).

## Discussion

The success of ASF control measures and passive surveillance depends on the willingness of hunters to implement them (2, 11, 14). It is therefore of utmost importance that the national and international control of ASF focuses on identifying motivations or obstacles to support control measures and passive surveillance and, if necessary, on increasing the willingness of hunters to participate in these measures actively. To achieve this, PE methods should more frequently be included to complement conventional epidemiological approaches, also in industrialized countries. By integrating key stakeholders, decisions can be made based on extended information from the everyday life of those, who are directly affected and involved. However, this also influences the decision-making process by adding new biases, which are present in most participatory studies.

In the studies analyzed here, a potential selection bias may have been present due to the recruitment process (11, 13). Inviting participants through hunting associations holds the danger of recruiting only hunters of the direct social network of the contact person, who may share a common opinion. In addition, it is possible that mainly hunters were recruited, who were highly communicative toward hunting organizations and authorities (28). In addition, contact by the Ministry may have resulted in a situation, where some hunters felt compelled or obliged to participate and others may have been deterred. However, the roughly equal number of participants in both countries suggests that this bias has probably been low. The willingness to participate was therefore generally present and there was no obvious indication that hunters felt compelled to become involved. The total number of 96 participants may question the representability of the results. However, theoretical saturation was found in both studies as described in Glaser et al. (29) and Guest et al. (30). As the results were largely similar in both studies, which included hunters with a very different social background, the participation bias and question of representability may be regarded as minor.

Although the procedures to be followed by both facilitators were identical, a complete consistency cannot be guaranteed. Skills that characterize a good facilitator to get the most unfiltered results in a discussion could not be conveyed in short training provided to the facilitators (31), who also lacked experience in conducting PE studies. Furthermore, the openness of the participants toward an employee of a university (Estonia) might differ from the attitude toward an employee of a national authority (Latvia). In addition, there is the possibility that certain opinions may have been expressed in Latvia, precisely because the authority organized and carried out the FGDs. It seems possible that the hunters wanted to keep or create a certain image when confronted with a representative of a state authority or to stimulate certain reactions by the authority. As a male employee working in ASF disease control was present in the Latvian FGDs for questions and misunderstandings, this may have influenced hunters' statements. However, the general overlap of the results suggests that this potential bias had little impact on the outcome.

Direct transcription in Latvia instead of recording in Estonia had the advantage that no further transcript had to be made from the audio recording. However, direct transcribing the contents of the FDGs might have led to a loss of information due to subjectivity, as it is very likely that not every spoken word was considered important, so that some statements could have been missed. The translation process of both transcripts into English might have caused some information loss (translation bias).

Diverging extraneous circumstances like substantial differences in ASF control, varying hunting structures and the biases discussed above prevent that a detailed statistical analysis adds value to the conclusions that can be drawn by a simple descriptive comparison. Moreover, several results were only available in a qualitative form, which made a statistical comparison not only extremely difficult, but also and not very telling. We therefore focused on the purely descriptive comparison. Despite these potential biases, the statements of the participants in Estonia and Latvia showed similarities. For some topics, almost identical statements were made. This does not only show the strong and similar opinions of the hunters, but also suggests that these biases can be regarded as minor.

The acceptance of working with stakeholders in the hunters' network strongly overlapped in both studies. This indicated relationships, which may be utilized and improved. Various possible co-operations (e.g., support from the army) should be discussed in advance with the hunters; otherwise they might feel not sufficiently respected in their main competence, i.e., hunting. It could be discussed, for example, that the army/police might only support carcass search and not hunting, which may subsequently lead to a higher acceptance of this measure by the hunters. Furthermore, the dissatisfaction with the small numbers of contacts with the research centers became obvious. This again supports the importance of communication, also with regard to scientific exchange before implementing measures. The differences in the networks of hunters in the two countries appeared to be small. The lack of mentioning various public stakeholders (e.g., animal welfare organizations, media) in Estonia compared to the ones mentioned in Latvia, could be explained by a different perception of the participants, who the relevant stakeholders were, or by a difference in the network of ASF control in Latvia.

The clear trend of acceptance of specific control measures was present in both countries, indicating a similar attitude of hunters, regardless of the individually implemented system of control measures. When interpreting the results, it must be taken into account that the two Baltic States are neighboring countries with a comparable recent history (32). Thus, the broad agreement in the perceptions and views of the hunters might be related to this neighborhood. To allow a more general statement about attitudes of hunters regarding ASF, it may be useful to implement the study in countries with more diverse geographical, historical and political background information.

Controlling ASF with hunting and increasing financial incentives for hunting is likely to find favor with hunters. Furthermore, the general acceptance of increasing incentives underlines the potential need of financial support for arising costs, such as equipment for biosecurity and transport. The same reasons given for not accepting fences (restricting other wildlife) and hunting female wild boar (morally contradictory to produce orphans in the farrowing season) reflect the common concerns of the hunting community and should be solved if these measures are to be implemented. Additionally, the high acceptance of vaccination and low acceptance of increased carcass search show how important scientific exchange is, especially on these specific topics to discuss effectiveness and in the case of vaccination availability (2, 7, 9, 27).

The fact that only in Latvia biosecurity during hunting was mentioned as a measure and only in Estonia several specific hunting methods were listed might show the different prioritization or awareness of control measures in the two countries. Biosecurity was mentioned in Estonia not before discussing passive surveillance and transporting carcasses. Thus, the awareness of hunters that biosecurity is appropriate in any handling of wild boar should be increased accordingly. However, it should also be considered that in Estonia, hunters just forgot to mention biosecurity as a control measure without any indication for the general perceived importance of biosecurity measures in Estonia.

The findings of Calba et al. (13) and Schulz et al. (11) that passive surveillance might not be highly accepted among hunters are supported by the perceptions of the hunters in the compared studies. Negative consequences such as increased workload, costs and time consumption were the focus in both countries. Reducing these hindering factors or even preventing them from occurring in the first place could significantly increase the acceptance of passive surveillance. All participants mentioned the same following approaches in this regard. Accordingly, the increase of financial support and the involvement of the army/police under the guidance of the hunters should be focused. In this respect, according to the participants, the emphasis should be on reducing the obligations of hunters. The implemented feedback systems seem to be sufficient, as additional detailed feedback was perceived not to be highly motivating in both countries. Thereby, increasing the details of feedback would only increase the workload for the veterinary laboratories without achieving higher participation rates in passive surveillance.

Despite the importance of eliminating negative consequences, Latvian hunters were more motivated by their moral obligation to participate in passive surveillance in order to contain ASF. This difference may have been caused by a potential bias of the observer from the Latvian authority. As mentioned before, the presence of the Latvian authority may have motivated the hunters to make statements, which make them look favorable. On the other side, the self-image of hunters in Latvia as workers for nature and wildlife may be different from that in Estonia, as passive surveillance was more often described as “hunters' duty” during FGDs in Latvia. Since the assessment of the motivating options was only comparative, it is possible that the perceived obligation of hunters has a similar status in Estonia, but the lack of financial support was regarded as more significant. These differences emphasize the need to communicate with hunters in each country individually and with regard to their specific views and concerns.

In summary, two main issues could be identified, which should be considered in efforts to improving cooperation with hunters and thus supporting the joint fight against ASF.

First, communication and cooperation with hunters should be increased, especially when it comes to the decision-making process. Communication should also include the dialogue with research centers. Hunters would like to become involved in scientific discussion. This was mentioned by all participants. This will ensure that they are informed about the most recent research results on ASF by the researches themselves. On the other side, through a two-way communication, disease control will benefit from the expert knowledge of hunters in implementing practical and successful control systems. In this context, workshops or training courses may largely support increased communication. These events could be very helpful to explain the reasons and the possible positive effects of measures to the hunters as executive stakeholders, especially regarding passive surveillance. Possible modifications of already implemented measures could also be communicated, discussed and adapted jointly, for example hunting female wild boars only in autumn and winter.

Secondly, loss of time and the increased workload are the main conflicting issues for hunters to contribute to passive surveillance. These issues could be addressed by having other external stakeholders supporting the hunters by taking over the collection and disposal of wild boar carcasses after a hunter has reported the finding. If this is not possible, financial incentives or compensations may be increased to cover costs and time.

This study describes hunters' opinions regarding passive surveillance of ASF and measures to control ASF in two EU member states affected by the disease. In essence, despite different systems of ASF control and the different hunting structures in the EU member states there was broad consensus on a large number of issues in the hunting communities of Latvia and Estonia. The results of this study may be incorporated with caution into future work on ASF control, as they only reflect the opinions of a single stakeholder group. Participatory studies including stakeholders involved in ASF surveillance and control other than hunters should also be conducted or these groups included.

## Data Availability Statement

The original contributions presented in the study are included in the article/Supplementary Material, further inquiries can be directed to the corresponding author/s.

## Author Contributions

NU drafted the manuscript for this comparing study. KS, CS-L, CS, and FC provided scientific input and background for the draft of the manuscript and revised it extensively. All authors contributed to the article and approved the submitted version.

## Conflict of Interest

The authors declare that the research was conducted in the absence of any commercial or financial relationships that could be construed as a potential conflict of interest.
